# Measurement and Data Correction of Channel Sampling Timing Walk-Off of Photonic Analog-to-Digital Converter in Signal Recovery

**DOI:** 10.3390/mi15020290

**Published:** 2024-02-19

**Authors:** Junli Qi, Xin Chen, Meicheng Fu, Hongyu Zhang, Wenjun Yi, Hui Zhang, Xiaoming Wei, Bo Shi, Tengfei Xu, Dezhi Su, Weihua Wang, Xiujian Li

**Affiliations:** 1 College of Science, National University of Defense Technology, Changsha 410073, China; qijunli_r@nudt.edu.cn (J.Q.); chenxin_cx@nudt.edu.cn (X.C.); fumeicheng10@nudt.edu.cn (M.F.); zhanghongyu@nudt.edu.cn (H.Z.); yiwenjun@nudt.edu.cn (W.Y.); 2 Institute of Plasma Physics, Hefei Institutes of Physical Sciences, Chinese Academy of Sciences, Hefei 230031, China; 3 Science Island Branch of Graduate School, University of Science and Technology of China, Hefei 230031, China; 4 Basic Department, Army Academy of Artillery and Air Defense, Hefei 230031, China; yhhzhhf@163.com (H.Z.); qjl16@mail.ustc.edu.cn (X.W.); shibo1982_1982@126.com (B.S.); 5 Beijing Institute of Systems Engineering and Information Control, Beijing 100071, China; xtf1201@126.com; 6 College of Basic Sciences for Aviation, Naval Aviation University, Yantai 264001, China; sudezhifun@163.com; 7 Institute of Physical Science and Information Technology, Anhui University, Hefei 230031, China

**Keywords:** photonic analog-to-digital converter, channel sampling, timing walk-off, data correction, signal recovery, microwave photonics

## Abstract

A two-channel, time–wavelength interleaved photonic analog-to-digital converter (PADC) system with a sampling rate of 10.4 GSa/s was established, and a concise method for measuring and data correcting the channel sampling timing walk-off of PADCs for signal recovery was proposed. The measurements show that for the two RF signals of *f*_1_ = 100 MHz and *f*_2_ = 200 MHz, the channel sampling timing walk-off was 12 sampling periods, which results in an ENOB = −0.1051 bits for the 100 MHz directly synthesized signal, while the ENOB improved up to 4.0136 bits using shift synthesis. In addition, the peak limit method (PLM) and normalization processing were introduced to reduce the impacts of signal peak jitter and power inconsistency between two channels, which further improve the ENOB of the 100 MHz signal up to 4.5668 bits. All signals were analyzed and discussed in both time and frequency domains. The 21.1 GHz signal was also collected and converted using the established two-channel PADC system with the data correction method, combining the PLM, normalization, and shift synthesis, showing that the ENOB increased from the initial −0.9181 to 4.1913 bits, which demonstrates that our method can be effectively used for signal recovery in channel-interleaved PADCs.

## 1. Introduction

Digital signals have advantages such as good stability and security, a strong anti-interference ability, and a robust transmission compared with analog signals. The key device for converting analog signals into digital signals is the analog-to-digital converter (ADC), which plays a very important role in the process of information processing, especially in the cutting-edge technology fields such as large bandwidth and ultra-high speed microwave signal processing systems, including radar, software radio, medical imaging, satellite communication, and so on [1,2,3,4,5,6]. However, due to limitations such as major time jitter, large high-frequency losses, and comparator ambiguity, traditional electric ADCs (EADCs) struggle to meet the requirements of a high sampling rate and large bandwidth.

The photonic ADC (PADC) based on ultra-short optical pulse trains has been demonstrated to be a powerful tool to overcome the bottleneck of EADCs, achieving an ultra-high sampling rate and ultra-wide bandwidth [7,8]. Anatol Khilo et al. have demonstrated that the photonic approach can deliver on its promise by digitizing a 41 GHz RF signal with an effective number of bits (ENOB) of 7.0 using a PADC. Taking advantage of photons in wideband processing and electrons in high-precision quantization, the photonic-sampled and electronic-quantized PADC has become one of the mainstream schemes [9,10,11,12,13,14,15]. For instance, Qingwei Wu et al. have realized a time–wavelength interleaved PADC with a 40 GSa/s sampling rate, and an RF signal of 2.5 GHz was sampled and quantized, achieving an ENOB of 3.45 bits [9]. Zhengkai Li et al. have implemented a dual channel PADC system with a 10 GHz repetition rate and a 19.8 fs time jitter pulse source, successfully sampling and quantifying 1.1 GHz and 36.3 GHz RF signals into ENOBs of 5.85 and 3.75 bits, respectively [14]. In these PADC schemes, RF signals are sampled through optical pulses and digitized through electronic quantizers, such as EADCs or oscilloscopes. Thanks to the high bandwidth and fast response of the electro-optic modulator, high-frequency RF signals can be directly loaded into high-speed optical sampling pulses smoothly, while the sampled pulses carrying RF signals need to be demultiplexed to multiple channels to adapt to a low sampling rate limited by the EADC. This kind of PADC is collectively nominated as a channel-interleaved PADC (CI-PADC), including the time interleaved PADC [16,17,18] and time-wavelength interleaved PADC [19,20,21,22,23].

The influence of channel mismatch on the performance of CI-PADCs has been widely studied, including clock timing mismatch, amplitude mismatch, pulse shape mismatch, photodetection bandwidth mismatch, and electronic aperture jitter mismatch [16,17,18,20,21,22,23,24]. And many mismatch correction methods and compensation algorithms, such as the spectral analysis and compensation method and fractional Fourier domain filtering, were proposed to eliminate spurious components and improve signal ENOBs. However, there are few research reports on the impact of a channel sampling timing misalignment caused by a fiber length misalignment or an electrical phase misalignment of optoelectronic or electronic devices such as a photoelectric detector (PD), EADC, RF lines, etc., on channel synchronization.

Herein, we established a two-channel, time–wavelength interleaved PADC system with a sampling rate of 10.4 GSa/s, and a concise method for measuring and correcting the channel sampling timing misalignment of PADCs in signal recovery was demonstrated. Two detection RF signals, *f*_1_ = 100 MHz and *f*_2_ = 200 MHz, were selected to measure the misalignment by finding the minimum root mean square error (MRMSE). When the data from channel 2 were shifted forward 12 positions, the MRMSEs of the two detection signals were found with the values of 44.4174 and 28.9738, respectively. Additionally, the peak limit method (PLM) and normalization processing were adopted to reduce the impacts of signal peak jitter and power inconsistency between the two channels. The signals collected from the two channels, as well as the corresponding direct synthesis signals and shift synthesis signals, were analyzed and discussed in both time and frequency domains. The ENOB of the signal synthesized directly from two channels of 100 MHz data was only −0.1051 bits, while it was improved up to 4.0136 bits after correcting the channel sampling timing misalignment. Further, the ENOB reached 4.5668 bits when the PLM and normalization were added. Finally, the established two-channel PADC system was used to detect the 21.1 GHz signal. The ENOB had increased from the initial −0.9181 to 4.1913 bits by the joint corrections of the PLM, normalization, and shift synthesis. Our proposed measurement and correction method can help achieve channel synchronization by controlling the fiber length of each channel in the hardware, as well as effectively recover the test signals in software data processing.

## 2. Methods and Simulations

**Figure 1:**
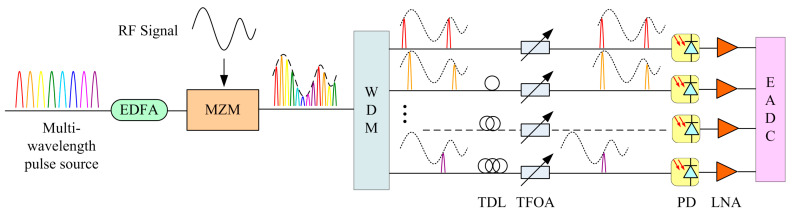
N-channel, time–wavelength interleaved PADC system sketch. EDFA, erbium-doped fiber amplifier; MZM, Mach–Zehnder modulator; WDM, wavelength division multiplexer; TDL, tunable delay line; TFOA, tunable fiber optic attenuator; PD, photoelectric detector; LPF, low-pass filter.

Figure 1 depicts the multi-channel, time–wavelength interleaved photonic analog-to-digital converter (PADC) system used for measurements. A multi-wavelength sampling pulse source with equal spacing can be generated by appropriately modulating a continuous-wave laser through cascaded intensity modulators and phase modulators [25]. An erbium-doped fiber amplifier (EDFA) is used to amplify the optical sampling pulse power loaded in the Mach–Zehnder modulator (MZM), ensuring its operation under good conditions. Meanwhile, the RF signals are loaded by the MZM. The sampling optical pulses modulated with RF signals are demultiplexed by a wavelength division multiplexer (WDM) and then converted into parallel N channels of single wavelength optical signals with a pulse frequency of *f_s_*, which equals to or is less than the sampling rate of the back-end electronic analog-to-digital converter (EADC), with the benefits of a mature production process and stable performance. Due to the possible delay changes of different wavelength channels after applying the WDM and other optical and electrical elements, the N-channel optical pulses are aligned with the EADC through a tunable delay line (TDL), making sure the EADC collects the peak values of optical pulses within one period, where *t_s_* = 1/*f_s_*. So, the delay between each channel is an integer multiple of *t_s_*. In addition, due to the different channel insertion losses caused by the WDM and other components, the optical intensity is relatively compensated through a tunable fiber optic attenuator (TFOA), which also ensures that the optical pulse intensity is located in the linear operating region of the PD. Then, the optical signal is converted into an electrical signal through the PD, amplified by a low-noise amplifier (LNA), keeping it in the optimal working state of the EADC.

### 2.1. Distortion of Misaligned Synthetic Signal

Due to the limited adjustment range of the TDL, the larger the adjustment range, the higher the price. In order to reduce costs and ensure that EADC can collect the peak value of the optical signal pulse, an adjustment range of 1–2 times *t_s_* can be selected. But when the delay of each channel exceeds 2 *t_s_*, it will be impossible to maintain real-time synchronization between each channel. At this point, the signal we obtain will be distorted and significantly different from the original signal.

Taking the PADC system with *N* = 4 and *f_s_* = 5.2 GHz as an example, the total sampling frequency *Fs* = *Nf_s_* = 20.8 GHz. It is assumed that the sampled RF signal is a normalized sine signal with a frequency of *f*_0_ = 100 MHz, i.e., V = sin (2*πf*_0_*t*). Further assuming that the first channel signal is synchronized with the original signal, the delays of channels 2–4 relative to the 1st channel is ∆*t*_2_ = 3*t_s_*, ∆*t*_3_ = −8*t_s_*, and ∆*t*_4_ = 5*t_s_*, respectively, where the positive sign represents leading, and the negative value represents lagging. The signals of each channel can be represented as follows:

(1) $$\left\{\begin{array}{l} V_{1}=\sin(2\pi \;f_{0}t_{1})\\ V_{2}=\sin\left[2\pi \;f_{0}(t_{2}+\Delta t_{2})\right]=\sin\left[2\pi \;f_{0}(t_{2}+3t_{s})\right]\\ V_{3}=\sin\left[2\pi \;f_{0}(t_{3}+\Delta t_{3})\right]=\sin\left[2\pi \;f_{0}(t_{3}-8t_{s})\right]\\ V_{4}=\sin\left[2\pi \;f_{0}(t_{4}+\Delta t_{4})\right]=\sin\left[2\pi \;f_{0}(t_{4}+5t_{s})\right] \end{array}\right.$$

in which *t*_1_–*t*_4_ are the time series in the original signal when the 4 channel signals are synchronized, which is a time vector. Taking *t*_1_ as an example, *t*_1_ = [*t*_11_, *t*_12_, … *t*_1*i*_, …, *t*_1*M*_], where *M* is the number of samples. The following is the relationship between them:

(2) $$t_{2i}-t_{1i}=t_{3i}-t_{2i}=t_{4i}-t_{3i}=t_{s}/N=1/(4f_{s})$$

**Figure 2:**
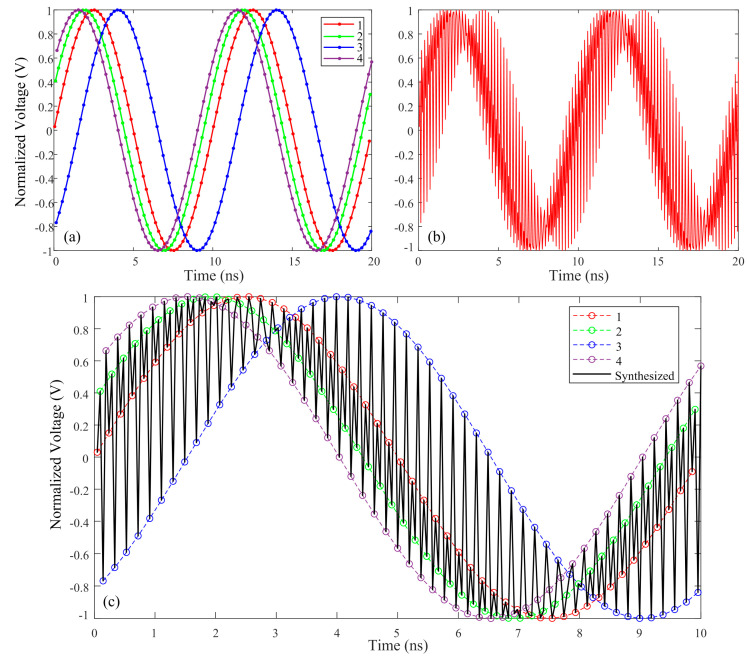
Four-channel signals collected by EADC and the directly synthesized signal. (**a**) 4-channel signals with different delays; (**b**) directly synthesized signal; (**c**) signal synthesis process.

The RF signals collected by the four channels of the EADC are shown in Figure 2a, and the actual original signal should overlap with the signal from channel 1. The signals of channels 1–4 are represented in red, green, blue, and purple, respectively, with solid dots representing the quantified values collected by the EADC. It is obvious that the signals of channels 2 and 4 are ahead of channel 1, while channel 3 lags behind channel 1. The signal directly synthesized according to the timing of the signals collected by the EADC is shown in Figure 2b, which shows significant distortion compared to the original signal. The detailed synthesis process is shown in Figure 2c. The solid circles in each channel represent the points collected by the EADC, and the dashed lines represent the fitted signals. The black solid line represents the signal directly synthesized by the four channels, which can be obtained by connecting them in chronological order.

**Figure 3:**
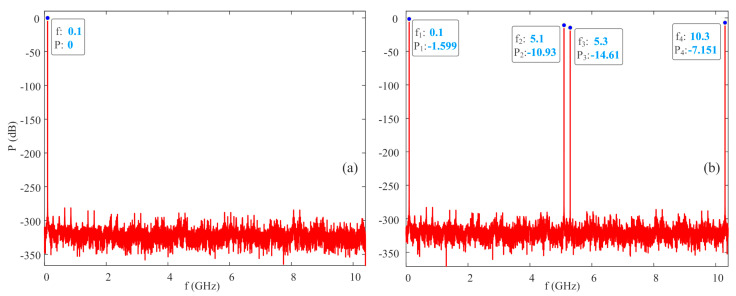
Spectrum information of original signal and directly synthesized signal. (**a**) Original signal; (**b**) directly synthesized signal.

In order to further illustrate the difference between the original signal and the directly synthesized signal, the effective number of bits (ENOB) and spectral information are calculated from the simulation, assuming that the original signal in the simulation is ideal and only constrained by the numerical accuracy of double-precision, floating-point calculations. The ENOB of the original signal can exceed 44 bits, while the ENOB of the directly synthesized signal is only 0.2918 bits. It can be found that there is only 100 MHz of spectrum information in the original signal from Figure 3a. In addition to the fundamental frequency spectrum of 100 MHz, there are also three other spectra in the directly synthesized signal: 5.1 GHz, 5.3 GHz, and 10.3 GHz in Figure 3b. From the labeled spectrum energy in Figure 3b, it can be seen that the power of each spectrum is relatively high, indicating that there is significant distortion in the directly synthesized signal.

### 2.2. Measurement and Correction of Misaligned Signals

As assumed in Section 2.1, the original RF signal to be tested is kept in phase synchronization with the signal collected in channel 1. A triangular interpolation can be performed on the 1st channel’s signal to achieve the N-fold expansion, i.e.,

(3) $$\left\{\begin{array}{l} V_{1}=\sin(2\pi \;f_{0}t_{1})\\ V_{2}^{\prime }=\sin(2\pi \;f_{0}t_{2}^{\prime })\\ V_{3}^{\prime }=\sin(2\pi \;f_{0}t_{3}^{\prime })\\ V_{4}^{\prime }=\sin(2\pi \;f_{0}t_{4}^{\prime }) \end{array}\right.$$

in which the following relationship between *t*_1_, *t*_2_′, *t*_3_′, and *t*_4_′ exists:

(4) $$t_{2i}^{\prime }-t_{1i}=t_{3i}^{\prime }-t_{2i}^{\prime }=t_{4i}^{\prime }-t_{3i}^{\prime }=1/(Nf_{s})$$

Subsequently, the signals collected by channels 2–4 of the EADC are shifted separately. Taking channel 2 as an example, assuming that after moving *k_2_* sampling periods, the measured values align closely with the corresponding interpolation, and the root mean square error between them will be minimized, that is, the MRMSE will be found. If *k*_2_ > 0, it indicates that channel 2 is leading. In order to maintain synchronization with channel 1, an increase in the delay is required, which means that channel 2 needs to move forward along the timeline, artificially increasing the delay amount, corresponding to the *V*_2_ sequence moving *k*_2_ positions to the right as a whole. Then, by removing the last *k*_2_ terms from *V*_2_ and subtracting the last (*M* − *k*_2_) terms of *V*_2_′, each term is squared, the average is calculated, and is then taken to the square root to obtain the MRMSE, i.e.,

(5) $$\begin{array}{cc} {\mathrm{MRMSE}}_{2} & =\sqrt{\left[{\left(V_{21}-V_{2(k_{2}+1)}^{\prime }\right)}^{2}+{\left(V_{22}-V_{2(k_{2}+2)}^{\prime }\right)}^{2}+\ldots +{\left(V_{2(M-k_{2})}-V_{2M}^{\prime }\right)}^{2}]/(M-k_{2}\right)}\\ & =\sqrt{\sum_{i=1}^{M-k_{2}}{\left(V_{2i}-V_{2(k_{2}+i)}^{\prime }\right)}^{2}/(M-k_{2})} \end{array}$$

**Figure 4:**
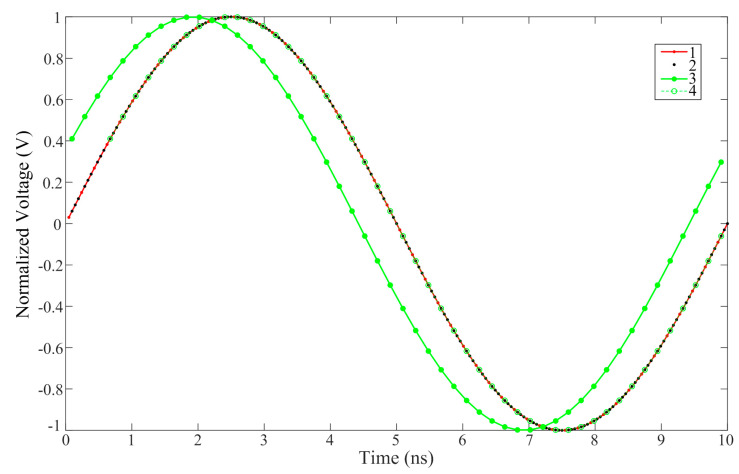
Interpolation of channel 1 and shift process of channel 2.

Assuming that *Z* is the smallest integer in the common multiple of *f_s_/f*_0_′, where *f*_0_′ is the frequency of the RF signal to be tested within the Nyquist bandwidth at an *fs* sampling rate, two *k*_2_ values can always be found between (−*Z*, *Z*), resulting in the MRMSE_2_. One value is positive, indicating that it needs to be shifted forward, and the other is negative, meaning a backward shift. The shift process is illustrated in Figure 4, where the red line represents the signal fitted to channel 1, the red solid points represent the values collected by the EADC of channel 1, the black solid points represent the (*N* − 1) times values that obtained through the trigonometric interpolation on channel 1 (excluding the values of channel 1), and the green line and solid points represent the fitted signal and the values collected by the EADC of channel 2, respectively. When shifted 3 positions forward, *V*_2_ is essentially aligned with the corresponding interpolation points of channel 1. At this moment, green dashed lines and circles are used to represent the signal and collected values of channel 2 after the shift, allowing for the calculation of the MRMSE. Similarly, when shifted backward by (*f_s_/f*_0_′ − 3) = 49 positions, i.e., *k*_2_ = −49, channel 2 also basically coincides with the interpolation points.

Since the delay amount of the PADC system remains constant regardless of the frequency of the signal being tested, substituting a new RF signal will result in two additional *k*_2_ values, with the unchanged *k*_2_ representing the actual delay amount. Similarly, channels 3 and 4 can also achieve their corresponding MRMSEs through appropriate shifts. Assuming *k*_3_ < 0 and *k*_4_ > 0, then

(6) $$\begin{array}{c} {\mathrm{MRMSE}}_{3}=\sqrt{\sum_{i=1}^{M+k_{3}}{\left(V_{3(i-k_{3})}-V_{3i}^{\prime }\right)}^{2}/(M+k_{3})}\\ {\mathrm{MRMSE}}_{4}=\sqrt{\sum_{i=1}^{M-k_{4}}{\left(V_{4i}-V_{4(k_{4}+i)}^{\prime }\right)}^{2}/(M-k_{4})} \end{array}$$

By using the same method mentioned above, the delay amounts *k*_3_ and *k*_4_ of the system can be obtained, and the PADC system can be corrected.

## 3. Experimental Results, Verification, and Discussion

To verify the correctness of the proposed correction method, we conducted measurements on a two-channel PADC system. As depicted in Figure 1, wavelengths of 1550.116 and 1540.577 nm are selected for these experiments, and the MZM modulator has a 3 dB bandwidth of 28 G to ensure the loading of broadband RF signals. The bandwidth of the selected PD is 10 GHz, with a built-in, low-noise amplifier (LNA). The EADC has a 3 dB bandwidth of 8 GHz and a sampling rate of up to 5.2 GSa/s. The adjustable fiber delay line TDL with an adjustment accuracy of less than 10 fs is selected within a range of 330 ps. For a sampling rate of 5.2 GSa/s, the sampling period is *t_s_* = 1/5.2 G = 192.308 ps, which ensures that the EADC can find the peak of the optical pulse within one period.

Two detection RF signals, i.e., *f*_1_ = 100 MHz and *f*_2_ = 200 MHz, were selected to measure the misalignment of the PADC system. Assuming that the refractive index n in the fiber core is *n* = 1.5, and the speed of light in vacuum is *c* = 3 × 10^8^ m/s, for the *f*_2_ = 200 MHz optical pulse signal, the fiber length that light propagates in one period is *L*_2_ = *c*/(*n*·*f*_2_) = 1 m, which ensures that the misalignment can be found within the 1 m fiber length range. For the *f*_1_ = 100 MHz optical pulse, the misalignment can be found within the *L*_1_ = 2 m range. In general, the fiber misalignment of our PADC system or the equivalent fiber length misalignment of the electrical phase misalignment from other optoelectronic or electronic devices (such as PD, EADC, RF lines, etc.) will not exceed 1 m. Therefore, these two detection RF signals can be used for determining the fiber length misalignment and correct it.

According to the misalignment correction method proposed in Section 2.2, the EADC uses a 5.2 GSa/s sampling rate to process the collected 35 K of data. Assuming channel 2 shifts *K*_f_ sampling periods forward or *K*_b_ periods backward, respectively, two MRMSEs will be obtained. For the two selected detection RF signals, the measurement results are shown in Table 1. That is, for the *f*_1_ = 100 MHz RF signal, when channel 2 moves forward by 12 positions, an MRMSE_f = 44.4174 will be obtained, and the maximum root mean square error obtained in this direction is 1087.30. When channel 2 moves backward 40 positions, an MRMSE_b = 45.3537 will be obtained, and the maximum root mean square error in this direction is 1087.32. For the same PADC system detecting the *f*_2_ = 200 MHz RF signal, when channel 2 shifts forward by 12 positions or backward by 14 positions, an MRMSE_f = 28.9738 and MRMSE_b = 30.6193 will be obtained, and the maximum root mean square errors obtained in both directions are 1169.10 and 1169.17, respectively. So, for the two-channel PADC system with a 5.2 GSa/s sampling rate of each EADC, the misalignment between channels is 12 sampling periods, and channel 2 needs to lag by 12 sampling periods to synchronize with channel 1. Thus, the PADC system can be improved by lengthening the fiber length corresponding to channel 2 by approximately 12 × (*c*/*n*) × *t*_s_, or shortening the fiber length of channel 1, which can synchronize the multi-channel PADC system. Alternatively, without changing the PADC system, here, the data should be phase shifted before directly synthesizing the original signal.

**Figure 5:**
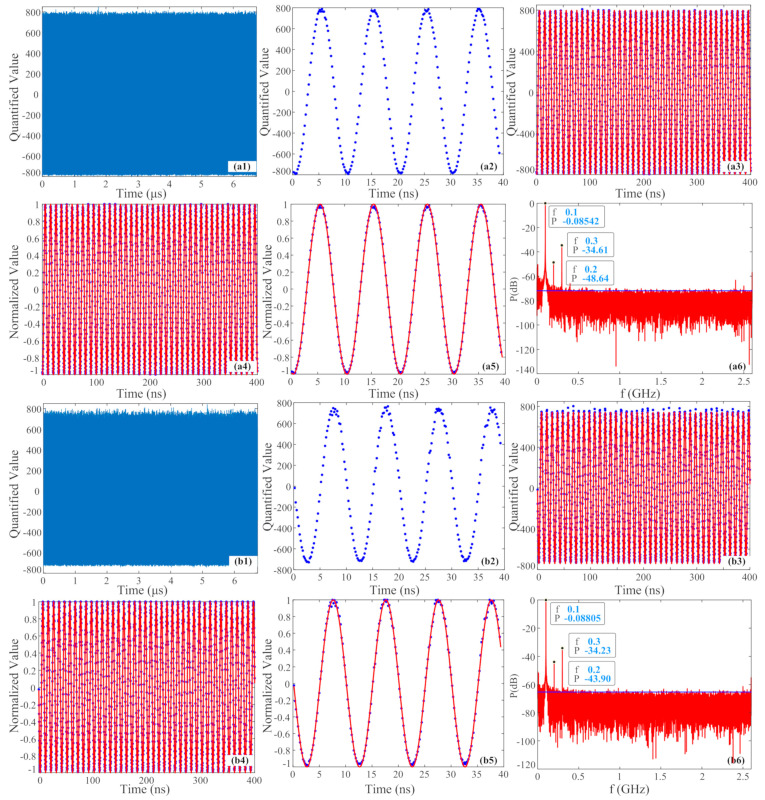
Two channel signals of 100 MHz collected by the PADC system. (**a**) Data of channel 1; (**b**) data of channel 2; 1–6: raw data, zoom-in data of first 40 ns, raw data and fitted signal of first 400 ns, peak-limited and normalized raw data and fitted signal of first 400 ns, zoom-in raw data and fitted signal of first 40 ns after the PLM and normalization, and normalized power spectrum.

The two channel signals of 100 MHz collected by the PADC system are shown in Figure 5. Figure 5(a1,b1) show the raw data collected by two channel EADCs, with the ENOBs of channel 1 and channel 2 being 4.9230 and 4.3192 bits and average peak-to-peak values of 1596 and 1484, respectively. It can be observed that the signal power of channel 2 is significantly lower than that of channel 1, and the maximum values of data collected by channel 2 have greater jitter, owing to the relatively higher PD noise in channel 2 and a corresponding lower ENOB. According to Figure 5(a2,b2), which show the zoomed-in raw data collected during the first 40 ns with more detailed information, we can find that the peak jitter of channel 2 is greater than that of channel 1, and the peak-to-peak value collected by channel 1 is greater, which means that the signal power of the same signal collected through two channels is inconsistent. In fact, as the average power consistency of the two channels has been modulated through the TFOA, the inconsistency here is caused by the instantaneity of signal acquisition and accidental jitter during photoelectric conversion by the PD. Figure 5(a3,b3) show the raw data collected during the first 400 ns and the corresponding fitted signals, depicted by solid blue dots and solid red lines [26]. By comparing the raw data with the fitted signal, it can be observed that the peak noise in channel 2 is actually higher.

In order to reduce the impact of peak jitter, we assign the collected signal values that exceed or fall below the maximum and minimum values of the fitted signal as the maximum and minimum values of the fitted signal, referred to as the peak limit method (PLM). Then, we normalize the signal to reduce the impact of inconsistent signal power between the two channels. Figure 5(a4,b4) depict the raw data and corresponding fitted triangular signal within the first 400 ns after applying the PLM and normalization processing. It can be observed that the signal peak jitter and power inconsistency between the two channels have been significantly reduced. Based on our calculations, the ENOBs of the two processed channels are 4.9241 and 4.3534 bits, respectively, which are higher than the original data values, and the ENOB of channel 2 has improved more significantly. Figure 5(a5,b5) show the normalized data and fitted signals for the first 40 ns, represented by solid blue dots and solid red lines. It can be observed that the intensity consistency of the two channels has been significantly improved, and the data from channel 1 match the fitted signal very well. However, the data from channel 2 not only experience significant jitter near the peak, but also have a poorer consistency with the fitted signal compared to channel 1, resulting in a lower ENOB. Figure 5(a6,b6) show the normalized power spectrum information. The top three frequencies and power information with the strongest power are marked in the figures. It can be observed that the strongest frequency component in both channels is 100 MHz, which is consistent with the detection signal frequency (which is the main frequency), and the second strongest frequencies are both 300 MHz. The mutual power difference of 100 MHz in both channels is 0.0263 dB and is −0.38 dB for 300 MHz, and the differences are all small. The main difference lies in the power of the third strongest frequency, with a difference of −4.74 dB. Except for the main frequency, the lower the power of the other frequencies, the smaller the nonlinear distortion, indicating a relatively large distortion in channel 2. In addition, the blue solid horizontal line in the figure indicates the average peak value of the noise base, which is about −73 dB and −67 dB, respectively, indicating that the noise in channel 2 is relatively large. Overall, through the PLM and normalization processing, the signal peak jitter and inconsistent power between the two channels can be reduced. Moreover, the nonlinear distortion and noise of the signals collected from channel 2 are relatively large.

**Figure 6:**
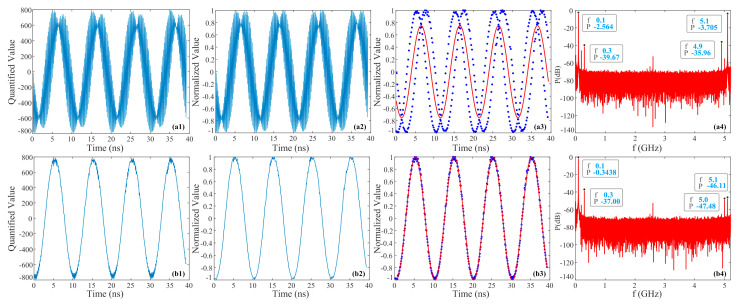
Experimental results of direct synthesis and shift synthesis of two channels’ data for 100 MHz. (**a**) Directly synthesized signal; (**b**) signal synthesized by splicing after shifting; 1–4: original synthesized signal, synthesized signal after the PLM and normalization, fitted synthesized signal after the PLM and normalization, and normalized power spectrum.

Figure 6 shows the signals from the direct synthesis and shift synthesis of the two channels’ data. Figure 6(a1) shows the signal synthesized directly within the first 40 ns of the two channels, and a significant intensity non-uniformity can be observed. Figure 6(a2) shows the result of the two channel signals being synthesized after the PLM and normalization processing, and it can be observed that their intensity non-uniformity has been significantly improved. Figure 6(a3) depicts the fitted signal according to the data in Figure 6(a2) by the main frequency of 100 MHz. The solid blue dots represent the directly synthesized data, and the solid red lines represent the fitted signal. It can be found that the collected data do not match the fitted signal very well, and the calculated ENOB is only −0.1051 bits. Figure 6(a4) illuminates the power spectrum information of the directly synthesized signal. The top four frequencies and corresponding power information with the strongest power are marked in the figure. Among them, 100 MHz and 5.1 GHz have the highest power, and the difference in power is not significant, so there will be two main frequencies. The next ones with higher power are 4.9 GHz and 300 MHz, so the nonlinear distortion of the directly synthesized signal is significant. In addition, it can be observed that the average peak value of the noise base is approximately −70 dB, which is the average value of the peaks of the noise base of the two channels. The result of synthesizing the data of channel 1 and that of channel 2 after shifting forward by 12 positions is shown in Figure 6(b1). It can be clearly seen from the time domain that it is a 100 MHz signal, and its ENOB is calculated to be 4.0136 bits. Compared with Figure 6(a1), it further verifies the effectiveness of using shift synthesis to recover the signal to be tested. Figure 6(b2) presents the synthesized result of the two channel signals being processed by the PLM and normalization, and then shifted. By comparison, it can be found that the processed signal has fewer peak spikes and smoother curves. Based on our calculation, its ENOB reached 4.5668 bits, which is significantly higher than that in Figure 6(b1). The signal quality has been significantly improved, further verifying the effectiveness of the PLM and normalization, which will be directly used in the subsequent signal processing. Figure 6(b3) shows the shift synthesized signal and corresponding fitted signal after applying the PLM and normalization processing, and it can be found that the processed data match the fitted signal very well. Figure 6(b4) shows the power spectrum information of the normalized shift synthesized signal. It can be observed that the Nyquist frequency has changed from a single channel 2.6 GHz to a dual channel synthesized 5.2 GHz. The figure indicates the top four frequencies with the strongest power and corresponding power information, with the highest power being the 100 MHz frequency, which is 36.66 dB higher than the three other frequencies, meaning there is only one main frequency. Therefore, the nonlinear distortion of the shift synthesized signal is small and can effectively recover the testing signal. In addition, the average peak noise level of the synthesized signal is also about −70 dB, which is related to the noise of the two channels.

**Figure 7:**
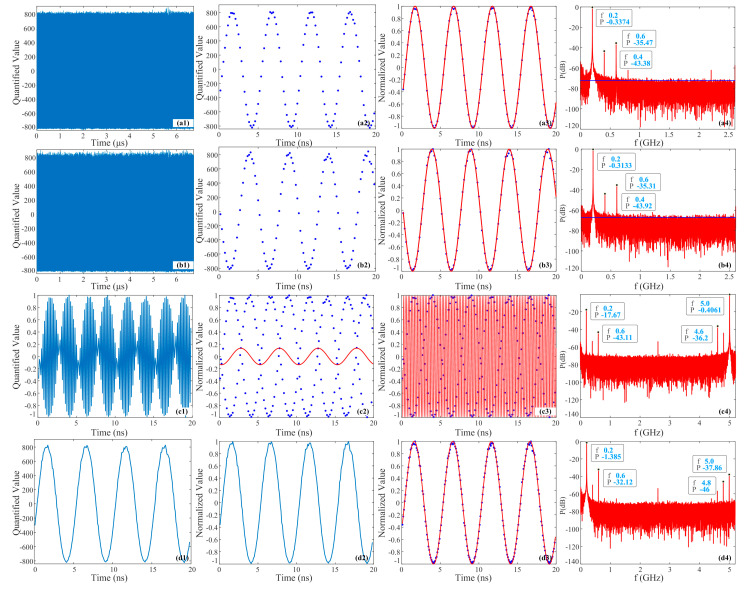
Experimental results of 200 MHz collected by the PADC system. (**a**–**d**) Data of channel 1, data of channel 2, synthesized signal directly, and synthesized signal after shift correction.

Figure 7 depicts the results of the PADC system collecting two channel signals at 200 MHz and synthesizing them directly and after the shift correction. Figure 7(a1,b1) display 35 K of raw data collected by two channels of EADCs. Overall, the signal peak jitter collected by channel 2 is relatively large. Based on our calculations, the ENOBs of channel 1 and channel 2 are 4.7417 and 4.3042 bits, respectively, with average peak-to-peak values of 1658 and 1660, indicating that the signal power of channel 2 is slightly higher, but the difference is quite small. Figure 7(a2,b2) depict the detailed raw data collected within the first 20 ns, making it easier to observe the peak jitter of channel 2. Figure 7(a3,b3) show the data and corresponding fitted trigonometric signals within the first 20 ns after implementing the PLM and normalization processing, represented by solid blue dots and solid red lines respectively. It can be observed that the peak jitter has been improved, and the original data match the fitted signal well. Based on our calculations, the ENOBs of the two channels are 4.7418 and 4.3229 bits, respectively, which are higher than the values of the original data, and the improvement in the ENOB of channel 2 is more significant. Figure 7(a4,b4) illuminate the normalized power spectrum information. The top three frequencies and power information with the strongest power are marked. It can be observed that the strongest frequency in both channels is 200 MHz, which is consistent with the test signal frequency, i.e., the main frequency. However, the power of channel 1 is slightly lower than that of channel 2, indicating that the main frequency intensity of channel 2 is relatively larger, but the difference is not significant, consistent with the findings in Figure 7(a1,b1). In addition, the power levels of the second- and third-strongest spectra are similar, but it is evident from the graph that the power heights of 400 MHz and 600 MHz relative to noise base in channel 1 are higher than those in channel 2, indicating that the noise base of channel 1 is relatively low. The blue solid horizontal lines in the graph indicate the average peak values of the noise base, which are approximately −74 dB and −66 dB, respectively. Therefore, compared to channel 1, the low ENOB in channel 2 is mainly caused by noise, and the impact of nonlinear distortion is relatively small.

Figure 7(c1) shows the result of directly synthesizing the two channels after using the PLM and normalization treatment within the first 20 ns, and it can be found that the intensity non-uniformity has been improved. Figure 7(c2,c3) depict the fitted signals according to the data in Figure 7(c1) by the frequencies 200 MHz and 5.0 GHz, respectively. The solid blue dots represent the directly synthesized data, and the solid red lines represent the fitted signal. It can be observed that the collected data do not match the fitted signal very well for Figure 7(c2), with the corresponding ENOB is only −3.1627 bits. For Figure 7(c3), the collected data are relatively consistent with the fitted signal. Based on our calculation, its ENOB reaches 2.5268 bits, indicating that the main frequency of the directly synthesized signal is no longer 200 MHz but rather, 5.0 GHz. Figure 7(c4) shows the power spectrum information of the directly synthesized signal after applying the PLM and normalization. The top four frequencies and power information with the strongest power are marked in the figure. Among them, the power at 5.0 GHz is the highest, followed by 200 MHz, which is 17.26 dB lower than the maximum power. Therefore, the data collected in Figure 7(c3) are more consistent with the fitted signal. The frequencies with higher powers are 4.6 GHz and 600 MHz. Thus, there is a significant nonlinear distortion for the directly synthesized signal. Additionally, it can be observed that the average peak value of the noise base is approximately −70 dB. Figure 7(d1) illustrates the result of channel 2 being shifted 12 positions forward and then combined with channel 1 to form a signal. It can be clearly seen from the time domain that it is a 200 MHz signal, and its ENOB is calculated to be 4.3445 bits, further verifying the effectiveness of using shift synthesis to recover the original signal. Figure 7(d2) shows the synthesized result of two channel signals being first processed by the PLM and normalization, and then shifted. Based on our calculation, the ENOB reaches 4.3831 bits, which is an improvement compared with Figure 7(d1), but not by much, because the power difference between the signals collected by the two channels is not significant, and the effect of normalization is relatively insignificant at this moment. Figure 7(d3) displays the shift synthesized signal and corresponding fitted signal after applying the PLM and normalization, and it can be found that the processed data match the fitted signal very well. Figure 7(d4) shows the power spectrum information of the normalized shift synthesized signal, which also indicates the top four frequencies and power information with the strongest power. The frequency with the highest power is 200 MHz, followed by 600 MHz, 5.0 GHz, and 4.8 GHz. The power of the main frequency is more than 30.73 dB higher than the other three frequencies, so the nonlinear distortion of the shift synthesized signal is small and can effectively recover the original signal. And it can be found that the average peak noise level of the synthesized signal is also about −70 dB.

**Figure 8:**
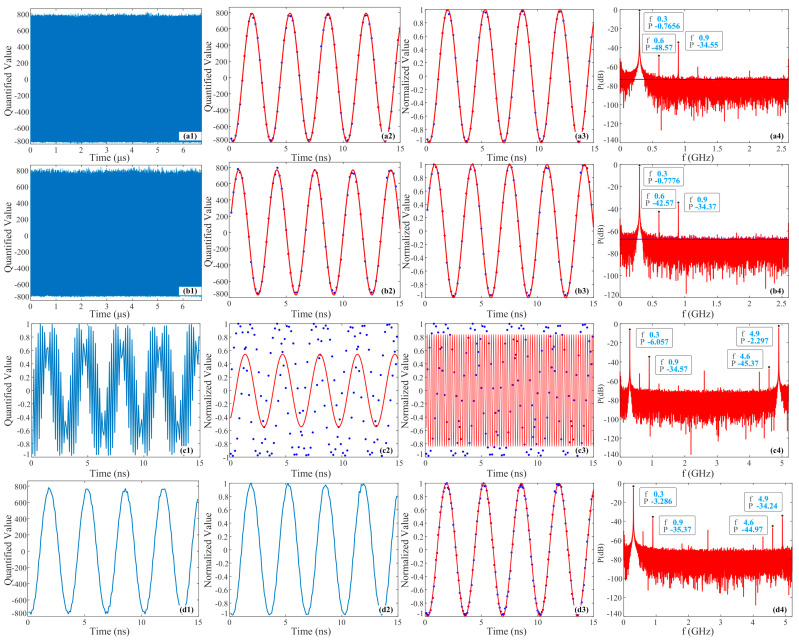
Experimental results of 21.1 GHz signal collected by the PADC system. (**a**–**d**) Data of channel 1, data of channel 2, synthesized signal directly, and synthesized signal after shift correction.

The results of the PADC system collecting 21.1 GHz RF signals are depicted in Figure 8. Figure 8a–d illustrate the results of channel 1, channel 2, direct synthesis, and shift corrected synthesis signals, similar to the corresponding relationship presented in Figure 7. According to the Nyquist sampling theorem, for sampling rates of 5.2 GSa/s and 10.4 GSa/s, the 21.1 GHz RF signal will be sub-converted to 300 MHz within the Nyquist bandwidth of 2.6 GHz and 5.2 GHz. Figure 8(a1,b1) display 35 K of raw data collected by two channels of EADCs. Overall, channel 2 exhibits a relatively large peak jitter. Based on our calculations, the ENOBs of channel 1 and channel 2 are 4.8677 and 4.3294 bits, respectively, with average peak-to-peak values of 1582 and 1538, indicating that channel 2 has a slightly lower signal power, albeit the difference is not substantial. Figure 8(a2,b2) present the zoomed-in raw signals collected within the first 15 ns. The solid blue dots represent the collected raw data, and the solid red lines represent the fitted triangular signals. It can be observed that channel 2 experiences greater peak jitter. Figure 8(a3,b3) display the data and corresponding fitted trigonometric signals within the first 15 ns following the PLM and normalization processing, represented by solid blue dots and solid red lines, respectively. It can be discerned that the signal peak jitter has been mitigated, and the original data align well with the fitting function. Based on our calculations, the ENOBs of the two channels are 4.8687 and 4.3575 bits, respectively, surpassing the values of the original data. Furthermore, the enhancement in channel 2’s ENOB is more pronounced, further verifying the effectiveness of the PLM and normalization method. Figure 8(a4,b4) illustrate the normalized power spectrum information. The top three frequencies and power information with the strongest power are highlighted in the figures. It can be observed that both channels exhibit the strongest power at 300 MHz, followed by 900 MHz. The power difference at the same frequency in both channels is small for the top two frequencies, but it reaches approximately 6 dB at 600 MHz, indicating that channel 2 experiences a comparatively significant nonlinear distortion. In addition, the blue solid horizontal line in the figure signifies the average peak value of the noise base, which is approximately −76 dB and −66 dB for each channel, indicating that channel 2 also possesses a relatively high level of noise.

Figure 8(c1) illustrates the result of directly synthesizing the two channels after impleneting the PLM and normalization processing within the first 15 ns, and it can be found that the intensity non-uniformity has been improved. Figure 8(c2,c3) depict the fitted signals based on the data in Figure 8(c1) at frequencies of 300 MHz and 4.9 GHz, respectively. The solid blue dots represent the directly synthesized data, and the solid red lines represent the fitted signal. However, it can be noted that the collected data do not match the fitted signal accurately, with corresponding ENOB values of only −0.9181 and 0.3285 bits, respectively. Figure 8(c4) shows the power spectrum information of the directly synthesized signal after using the PLM and normalization. The top four frequencies and power information with the strongest power are marked in the figure, with 4.9 GHz having the highest power, followed by 300 MHz. Therefore, the fitting effect of 4.9 GHz is relatively better than that of 300 MHz, with a power difference of 3.76 dB between the two frequencies. Nevertheless, due to the insignificant difference and the unclear main frequency, the signals fitted with them do not match the collected data very well. The next higher power components are 900 MHz and 4.6 GHz. Thus, the nonlinear distortion of the directly synthesized signal is significant. By observation, it can be found that the average peak of the noise base is about −71 dB. Figure 8(d1) presents the result of channel 2 being shifted 12 positions forward and then combined with channel 1 to synthesize a signal. The calculated ENOB is 4.1184 bits, further substantiating the effectiveness of using shift synthesis to recover the original signal compared to Figure 8(c1). Figure 8(d2) shows the synthesized result of two channel signals being processed by the PLM and normalization, and then shifted. The ENOB reaches 4.1913 bits, which is slightly higher than that without using the PLM and normalization processing. However, the increase is not significant, as there is no significant power difference between the signals collected by the two channels. In addition, the ENOB of the synthesized signal is reduced compared to both channel 1 and channel 2 due to the inaccurate alignment of pulse peaks collected by the two channel EADCs, which has a greater impact on high-frequency synthesized signals. The next step will be to calibrate it using the FROG method to achieve a precise alignment between the pulse peaks and EADC [25]. Figure 8(d3) demonstrates the shift synthesized signal and corresponding fitted signal after applying the PLM and normalization, wherein the processed data match the fitted signal very well. Figure 8(d4) shows the power spectrum information of the normalized shift synthesized signal, indicating the top four frequencies and power information with the strongest power. The frequency corresponding to the highest power is 300 MHz, followed by 4.9 GHz, 900 MHz, and 4.6 GHz. The power of the main frequency is higher than the other three frequencies by more than 30.95 dB, but the maximum power is only −3.286 dB, which is relatively small compared to Figure 7(d4), suggesting that there are more spectral components and a larger proportion of power. For instance, the difference between the second and third power is only 1.13 dB, resulting in a slightly lower ENOB for the synthesized signal compared to channel 1 and channel 2. Additionally, the average peak noise of the synthesized signal is also approximately −71 dB, which is related to the noise of the two channels.

## 4. Conclusions

In summary, we have developed a two-channel, time–wavelength interleaved PADC system with a sampling rate of 10.4 GSa/s, and demonstrated a concise method to measure and correct channel sampling timing walk-off of a PADC in signal recovery. Two detection RF signals of *f*_1_ = 100 MHz and *f*_2_ = 200 MHz were selected for measuring the channel sampling timing misalignment by finding the MRMSEs. By shifting the data from channel 2 forward by 12 positions, we obtained MRMSE values of 44.4174 and 28.9738 for the two detection signals, respectively. Additionally, we employed the PLM and normalization processing to mitigate the impact of signal peak jitter and power inconsistency between the channels. The ENOB of the directly synthesized signal from two channels of 100 MHz data was only −0.1051 bits, while it improved up to 4.0136 bits after correcting the channel delay misalignment. Furthermore, the ENOB reached 4.5668 bits when the PLM and normalization were added. Through the joint correction of the PLM, normalization, and shift synthesis, the ENOB of the 200 MHz signal increased from the −3.1627 bits to 4.3831 bits. Finally, the established two-channel PADC system was applied to detect the 21.1 GHz signal. The ENOB had increased from the initial −0.9181 to 4.1913 bits using our correction method. The signals collected from the two channels, as well as the corresponding direct synthesis signals and shift synthesis signals, were analyzed and discussed in both the time and frequency domains. The experimental results have demonstrated that our proposed method can effectively recover the test signal, being immune to channel delay misalignment.

## Figures and Tables

**Table 1:** Measured MRMSEs and corresponding shift amounts of two detection RF signals.

*f* (MHz)	*k*_f_	MRMSE_f	*k*_b_	MRMSE_b
100	12	44.4174	−40	45.3537
200	12	28.9738	−14	30.6193

## Data Availability

Data are contained within the article.

## References

[1] Yao J., Capmany J. (2022). Microwave photonics. Sci. China Inf. Sci..

[2] Marpaung D., Yao J., Capmany J. (2019). Integrated microwave photonics. Nat. Photon..

[3] Ghelfi P., Laghezza F., Scotti F., Serafino G., Capria A., Pinna S., Onori D., Porzi C., Scaffardi M., Malacarne A. (2014). A fully photonics-based coherent radar system. Nature.

[4] Liu C., Xie Q., Wang R., Yang J., Ma W., Li W., Wu Y. (2023). Microwave photonic radar system with improved SNR performance utilizing optical resonant amplification and random Fourier coefficient waveforms. Opt. Express.

[5] Araujo T., Dinis R. (2007). Analytical Evaluation and Optimization of the Analog-to-Digital Converter in Software Radio Architectures. IEEE Trans. Veh. Technol..

[6] Coskun A., Cetinsel S., Kale I., Hughes R., Angeletti P., Ernst C. (2023). Digital Prototype Filter Alternatives for Processing Frequency-Stacked Mobile Subbands Deploying a Single Adc for Beamforming Satellites. IEEE Trans. Aerosp. Electron. Syst..

[7] Valley G.C. (2007). Photonic analog-to-digital converters. Opt. Express.

[8] Khilo A., Spector S.J., Grein M.E., Nejadmalayeri A.H., Holzwarth C.W., Sander M.Y., Dahlem M.S., Peng M.Y., Geis M.W., DiLello N.A. (2012). Photonic ADC: Overcoming the bottleneck of electronic jitter. Opt. Express.

[9] Wu Q., Zhang H., Peng Y., Fu X., Yao M. (2009). 40GS/s Optical analog-to-digital conversion system and its improvement. Opt. Express.

[10] Ma Y., Liang D., Peng D., Zhang Z., Zhang Y., Zhang S., Liu Y. (2017). Broadband high-resolution microwave frequency measurement based on low-speed photonic analog-to-digital converters. Opt. Express.

[11] Yang G., Zou W., Yuan Y., Chen J. (2018). Wideband signal detection based on high-speed photonicanalog-to-digital converter. Chin. Opt. Lett..

[12] Xu S., Zou X., Ma B., Chen J., Yu L., Zou W. (2019). Deep-learning-powered photonic analog-to-digital conversion. Light Sci. Appl..

[13] Zheng K., Zou W., Yu L., Qian N., Chen J. (2020). Stability optimization of channel-interleaved photonic analog-to-digital converter by extracting of dual-output photonic demultiplexing. Chin. Opt. Lett..

[14] Li Z., Wang X., Zhang Y., Shang C., Lyu W., Lyu Y., Zeng C., Zhang Z., Zhang S., Li H. (2022). Photonic sampling analog-to-digital conversion based on time and wavelength interleaved ultra-short optical pulse train generated by using monolithic integrated LNOI intensity and phase modulator. Opt. Express.

[15] Li Z., Tian H., Lyu W., Zhang Y., Gao F., Xu Z., Zhang L., Zhang Z., Zhang S., Li H. (2023). Instantaneous bandwidth expansion of photonic sampling analog-to-digital conversion for linear frequency modulation waveforms based on up-sampling and fractional Fourier transform signal processing. Opt. Express.

[16] Jin Z., Wu G., Wang C., Chen J. (2018). Mismatches analysis based on channel response and an amplitude correction method for time interleaved photonic analog-to-digital converters. Opt. Express.

[17] Qian N., Yu L., Chen J., Zou W. (2020). Influence of the Demultiplexer on Channel-Interleaved Photonic Analog-to-Digital Converters. IEEE Photonics J..

[18] Qian N., Zhang L., Chen J., Zou W. (2021). Characterization of the Frequency Response of Channel-Interleaved Photonic ADCs Based on the Optical Time-Division Demultiplexer. IEEE Photonics J..

[19] Wu G., Li S., Li X., Chen J. (2010). 18 wavelengths 83.9Gs/s optical sampling clock for photonic A/D converters. Opt. Express.

[20] Yang G., Zou W., Li X., Chen J. (2015). Theoretical and experimental analysis of channel mismatch in time-wavelength interleaved optical clock based on mode-locked laser. Opt. Express.

[21] Yang G., Zou W., Yu L., Wu K., Chen J. (2016). Compensation of multi-channel mismatches in high-speed high-resolution photonic analog-to-digital converter. Opt. Express.

[22] Yang G., Zou W., Yu L., Chen J. (2018). Influence of the sampling clock pulse shape mismatch on channel-interleaved photonic analog-to-digital conversion. Opt. Lett..

[23] Xu Y., Li S., Xue X., Xiao X., Zheng X., Zhou B. (2019). An Interleaved Broadband Photonic ADC Immune to Channel Mismatches Capable for High-Speed Radar Imaging. IEEE Photonics J..

[24] Yang G., Zou W., Yu L., Qian N., Chen J. (2019). Investigation of electronic aperture jitter effect in channel-interleaved photonic analog-to-digital converter. Opt. Express.

[25] Zhang H., Fu M., Chen X., Qi J., Yi W., Zhang Y., Zhang Y., Xu Y., Li X. (2023). Highly precise timing alignment of multi-wavelength interleaved cavity-less pulse sources with FROG. Opt. Express.

[26] Qi J., Chen X., Fu M., Zhang H., Yi W., Xu T., Su D., Zhang H., Wei X., Shi B. (2023). Effects of Optical Sampling Pulse Power, RF Power, and Electronic Back-End Bandwidth on the Performance of Photonic Analog-to-Digital Converter. Micromachines.

